# The value of net influx constant based on FDG PET/CT dynamic imaging in the differential diagnosis of metastatic from non-metastatic lymph nodes in lung cancer

**DOI:** 10.1007/s12149-024-01964-y

**Published:** 2024-07-29

**Authors:** Xieraili Wumener, Yarong Zhang, Zihan Zang, Xiaoxing Ye, Jiuhui Zhao, Jun Zhao, Ying Liang

**Affiliations:** 1 Department of Graduate School, Dalian Medical University, Dalian, China; 2 Department of Nuclear Medicine, National Cancer Center/National Clinical Research Center for Cancer/Cancer Hospital and Shenzhen Hospital, Chinese Academy of Medical Sciences and Peking Union Medical College/Shenzhen Clinical Research Center for Cancer, Shenzhen, China; 3 Shenzhen Middle School, Shenzhen, China; 4 Department of Pathology, National Cancer Center/National Clinical Research Center for Cancer/Cancer Hospital and Shenzhen Hospital, Chinese Academy of Medical Sciences and Peking Union Medical College/Shenzhen Clinical Research Center for Cancer, Shenzhen, China; 5 Department of Nuclear Medicine, Shanghai East Hospital Tongji University, Shanghai, China

**Keywords:** Dynamic imaging, PET/CT, ^18^F-FDG, Lung cancer, Net influx rate, Lymph node

## Abstract

**Objectives:**

This study aims to evaluate the value of the dynamic and static quantitative metabolic parameters derived from ^18^F-fluorodeoxyglucose (FDG)–positron emission tomography/CT (PET/CT) in the differential diagnosis of metastatic from non-metastatic lymph nodes (LNs) in lung cancer and to validate them based on the results of a previous study.

**Methods:**

One hundred and twenty-one patients with lung nodules or masses detected on chest CT scan underwent ^18^F-FDG PET/CT dynamic + static imaging with informed consent. A retrospective collection of 126 LNs in 37 patients with lung cancer was pathologically confirmed. Static image analysis parameters include LN-SUV_max_ and LN-SUV_max_/primary tumor SUV_max_ (LN-SUV_max_/PT-SUV_max_). Dynamic metabolic parameters including the net influx rate (*K*_i_) and the surrogate of perfusion (*K*_1_) and of each LN were obtained by applying the irreversible two-tissue compartment model using in-house Matlab software. *K*_i_/*K*_1_ was then calculated as a separate marker. Based on the pathological findings, we divided into a metastatic group and a non-metastatic group. The *χ*^2^ test was used to evaluate the agreement of the individual and combined diagnosis of each metabolic parameter with the gold standard. The receiver-operating characteristic (ROC) analysis was performed for each parameter to determine the diagnostic efficacy in differentiating non-metastatic from metastatic LNs with high FDG-avid. *P* < 0.05 was considered statistically significant.

**Results:**

Among the 126 FDG-avid LNs confirmed by pathology, 70 LNs were metastatic, and 56 LNs were non-metastatic. For ROC analysis, in separate assays, the dynamic metabolic parameter *K*_i_ [sensitivity (SEN) of 84.30%, specificity (SPE) of 94.60%, accuracy of 88.89%, and AUC of 0.895] had a better diagnostic value than the static metabolic parameter SUV_max_ (SEN of 82.90%, SPE of 62.50%, accuracy of 74.60%, and AUC of 0.727) in differentiating between metastatic from non-metastatic LNs groups, respectively. In the combined diagnosis group, the combined SUV_max_ + *K*_i_ diagnosis had a better diagnostic value in the differential diagnosis of metastatic from non-metastatic LNs, with SEN, SPE, accuracy, and AUC of 84.3%, 94.6%, 88.89%, and 0.907, respectively.

**Conclusions:**

When the cutoff value of *K*_i_ was 0.022 ml/g/min, it had a high diagnostic value in the differential diagnosis between metastasis and non-metastasis in FDG-avid LNs of lung cancer, especially in improving the specificity. The combination of SUV_max_ and *K*_i_ is expected to be a reliable metabolic parameter for N-staging of lung cancer.

## Introduction

Lung cancer is the leading cause of cancer-related death in both men and women worldwide. In China, the mortality rate is 30%, which ranks first [1]. In recent years, morbidity and mortality have been increasing in China [1]. For patients with lung cancer, accurate N-staging is critical for developing individualized treatment plans and predicting prognosis [2]. In addition to the loss of surgical options for stage N3 patients, the 5-year survival rate is reduced to 6% [3]. Therefore, improving the accuracy of lung cancer N-staging to reduce the false-positive rate is one of the issues that has attracted clinical attention.

^18^F-fluorodeoxyglucose (FDG)–positron emission tomography/computed tomography (PET/CT) plays an important role in the differential diagnosis, staging, response assessment, and prognosis of lung cancer. The sensitivity and specificity of FDG PET/CT in the differential diagnosis and accurate staging of lung cancer are limited. Because FDG is not a tumor-specific imaging agent, the standard uptake value (SUV) is affected by a variety of factors [4, 5]. In China, as an endemic region for endemic infectious diseases, some benign lung diseases such as tuberculosis, infections, and inflammatory and granulomatous diseases cause FDG-avid, leading to a decrease in the specificity of FDG PET/CT in N-staging of lung cancer [6–9]. In recent years, the use of dynamic FDG PET/CT (dPET/CT) imaging in oncology has received much attention. Dynamic metabolic parameters, such as net influx rate (*K*_i_) and tumor blood flow (*K*_1_), obtained based on the two-tissue irreversible compartment model approach, better describe the different metabolic stages of FDG and thus reflect the pathophysiological mechanisms of the disease [10, 11].

Previously, we conducted a series of studies related to the clinical application of dPET/CT in lung cancer [12–15]. In our preliminary study [12–15], we investigated the clinical value of dPET/CT in the differential diagnosis, N-staging, and epidermal growth factor receptor (EGFR) status prediction of lung cancer. The results of our preliminary study showed that the dynamic metabolic parameter Ki has a better differential diagnostic value in lung cancer differential diagnosis (cutoff value of 0.0250 ml/g/min) and EGFR status prediction (cutoff value of 0.0350 ml/g/min), especially improved specificity [13]. Of particular note, in a previous study [12], we compared the value of static metabolic parameters [SUV_max_, lymph node (LN)–SUV_max_/primary tumor (PT)–SUV_max_] and dynamically visualized metabolic parameters (*K*_i_ and *K*_i_/*K*_1_) in metastatic and non-metastatic FDG-avid LNs of lung cancer. We tentatively concluded that the dynamic metabolic parameters *K*_i_ and *K*_i_/*K*_1_ with high specificity at cutoff values of 0.022 ml/g/min and 0.093, respectively (specificity of 0.918 and 0.776, and AUCs of 0.672 and 0.673, respectively) were able to better discriminate the metastatic and non-metastatic LNs from the FDG-avid LNs. For static metabolic parameters, SUV_max_ and LN-SUV_max_/PT-SUV_max_ showed high sensitivity (0.826 and 0.999) at cutoff values of 4.050 and 0.236, respectively, but the specificity was suboptimal (specificity 0.388 and 0.204, and AUC 0.596 and 0.566, respectively).

To our knowledge, there are fewer previous relevant studies on the cutoff value of _i_. Based on our previous studies [12–15], we found that although *K*_i_ has good specificity in differential diagnosis, the sensitivity is not very reasonable. Therefore, in this study, we further investigated the clinical value*K* of (SUV_max_ and LN-SUV_max_/PT-SUV_max_) and dynamic metabolic parameters (*K*_i_ and *K*_i_/*K*_1_) in the differential diagnosis of FDG-avid LNs in lung cancer based on the cutoff value in our previous findings. The clinical value of each metabolic parameter in single or combined detection was investigated, and the results of the preliminary study were validated.

## Materials and methods

### Patients

The study was approved by the Ethics Committee of Cancer Hospital and Shenzhen Hospital, Chinese Academy of Medical Sciences (KYLH2022-1), and all patients signed a written informed consent before FDG PET/CT imaging.

A total of 121 patients underwent dPET/CT (chest, 65 min) + static FDG PET/CT imaging (sPET/CT, whole body, 10–20 min) from April 2022 to August 2023. All patients were found to have pulmonary nodules/masses on chest CT scans and were not receiving anti-inflammatory or anti-tumor therapy.

From the 121 patients, we retrospectively collected 126 FDG-avid LNs from 37 lung cancer patients to perform the present study. All 37 included patients enrolled had pathologically confirmed lung cancer. Postoperative pathology and/or puncture biopsy pathology confirmed 126 FDG-avid LNs as metastatic or non-metastatic LNs. The interval between the FDG PET/CT scan and the pathology results was less than 2 weeks.

Patients' FDG PET/CT scan characteristics of patients were collected, including PT-SUV_max_, FDG-avid LN long diameter, short diameter, LN-SUV_max_, and dynamic metabolic parameter values (including *K*_i_ and *K*_1_). Clinical information about the patients was also collected, including age, sex, type of primary lesion pathology, LN pathology results, and TNM staging [16]. LN locations were categorized according to the International Association for the Study of Lung Cancer LN map into the mediastinal region (zones 1–9) and the pulmonary hilar region (zones 10–12) [17]. The LNs enrolled in both dPET/CT and sPET/C corresponded to postoperative pathologic findings and or puncture biopsy sites and results. Finally, the results of the N-staging in dPET/CT and sPET/CT were based on the eighth edition of the TNM lung cancer classification [16].

### PET/CT data acquisition, reconstruction, and analysis

**Fig. 1:**
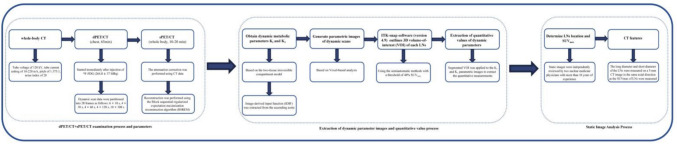
dPET/CT + sPET/CT scans examination procedure and data processing

Figure 1 shows the dPET/CT + sPET/CT examination process, data acquisition, image reconstruction, and metabolic parameter acquisition for the patient. All scans were performed in a Discovery MI PET/CT (GE Healthcare, Milwaukee, USA).

Dynamic parameters of *K*_i_ and *K*_1_ were then calculated and obtained based on the two-tissue irreversible compartment model. *K*_i_/*K*_1_ was computed subsequently as a separate marker. The image-derived input function (IDIF) was extracted from the ascending aorta by drawing a 10-mm-diameter ROI on six consecutive slices in an image obtained by combining early time frames (0–60 s), where the effects of motion and partial volume are less pronounced than in the left ventricle. In addition, this study did not account for differences in blood and plasma uptake. In this model, we assumed unidirectional uptake of ^18^F-FDG (i.e., *k*4 = 0) with irreversible trapping in the tissue as ^18^F-FDG-6-PO4 [18]. Parametric images of each dynamic scan were generated using voxel-based analysis. Due to the large number of voxels in a whole-body image, the Lawson–Hanson non-negative least-squares algorithm was used to solve a linearized problem instead of the conventional non-linear one [19].

### Pathological diagnosis

In this study, postoperative pathology and/or puncture biopsy pathology were the gold standard for follow-up.

All puncture and/or postoperative specimens were fixed in formalin, dehydrated, and embedded in paraffin. Four-micron sections of each tissue were stained with hematoxylin and eosin (H&E) and immunohistochemistry. The diagnosis was based on microscopic appearance and immunohistochemical results. The diagnosis was made independently by two experienced pathologists. In case of disagreement, the diagnosis was clarified after a full departmental discussion.

### Statistical analysis

Continuous variables were reported as the median and interquartile range. Categorical variables were described as number and frequency. The *χ*^2^ test was used to evaluate the agreement of the individual and combined diagnosis of each metabolic parameter with the gold standard. The receiver-operating characteristic (ROC) analysis was performed for each parameter to determine the diagnostic efficacy in differentiating non-metastatic from metastatic LNs with high FDG-avid. The difference in the area under the curve (AUC) was determined by Delong’s test. A *P* value of less than 0.05 was considered statistically significant. All statistical analyses were performed with R statistical software (version 4.1.1).

## Results

### Patients and LN characteristics

Patient and LN characteristics are shown in Table 1. Among the 37 patients who underwent dPET/CT + sPET/CT scans, the mean age was 59.95 (59.95 ± 11.44) years, and the number of male and female patients was 24 (64.87%) and 13 (35.13%), respectively. Among the 126 LNs that were pathologically confirmed, 56 (44.44%) LNs were non-metastatic and 70 (55.56%) LNs were metastatic.

**Table 1:** Characteristics of the patients and LNs

Characteristic	Distribution
*Sex* Male	24 (64.87%)
*Age (years)* Mean ± SD	59.95 (± 11.44)
*Lobar distribution of the primary tumor (N)*	37
RUL/RML/RLL	10 (27.03%)/3 (8.11%)/4 (10.81%)
LUL/LLL	12 (32.43%)/8 (21.62%)
*Histopathological type of the primary tumor (N)*	37
Adenocarcinoma	26 (70.27%)
Squamous cell carcinoma	5 (13.51%)
Small cell carcinoma	5 (13.51%)
pleomorphic carcinoma	1 (2.70%)
*Histopathological type of the LNs (N)*	126
Non-metastatic group	56 (44.44%)
Metastatic group	70 (55.56%)

*SD* standard deviation, *RUL* right upper lobe, *RML* right middle lobe, *RLL* right lower lobe, *LUL* left upper lobe, *LLL* left lower lobe

### Primary tumors and FDG-avid LNs ^18^F-FDG PET/CT characteristics summary

Among the 37 patients, the average SUV_max_ of the primary tumor was 10.95 (10.95 ± 4.48). In the metastatic LN_S_ group (*N* = 70), the ranges of long diameter, short diameter, SUV_max_, LN-SUV_max_/PT-SUV_max_, *K*_i,_ and *K*_i_/*K*_1_ were 1.95 cm [1.43;2.55], 1.30 cm [1.10;1.80], 7.75 [5.85;12.38], 0.69 [0.53;1.08], 0.023 [0.024;0.050] ml/g/min, and 0.295 [0.138;0.478], respectively. In the non-metastatic LN_S_ group (*N* = 56), the ranges of long diameter and short diameter, SUV_max_, LN-SUV_max_/PT-SUV_max_, *K*_i_, and *K*_i_/*K*_1_, were 1.30 cm [1.18;1.50], 1.00 cm [0.90;1.10], 3.50 [2.78;4.63], 0.45 [0.28;4.63], 0.012 [0.009;00016] ml/g/min, and 0.056 [0.0248;0.146], respectively.

### Diagnostic value of individual and combined tests for each metabolic parameter

**Fig. 2:**
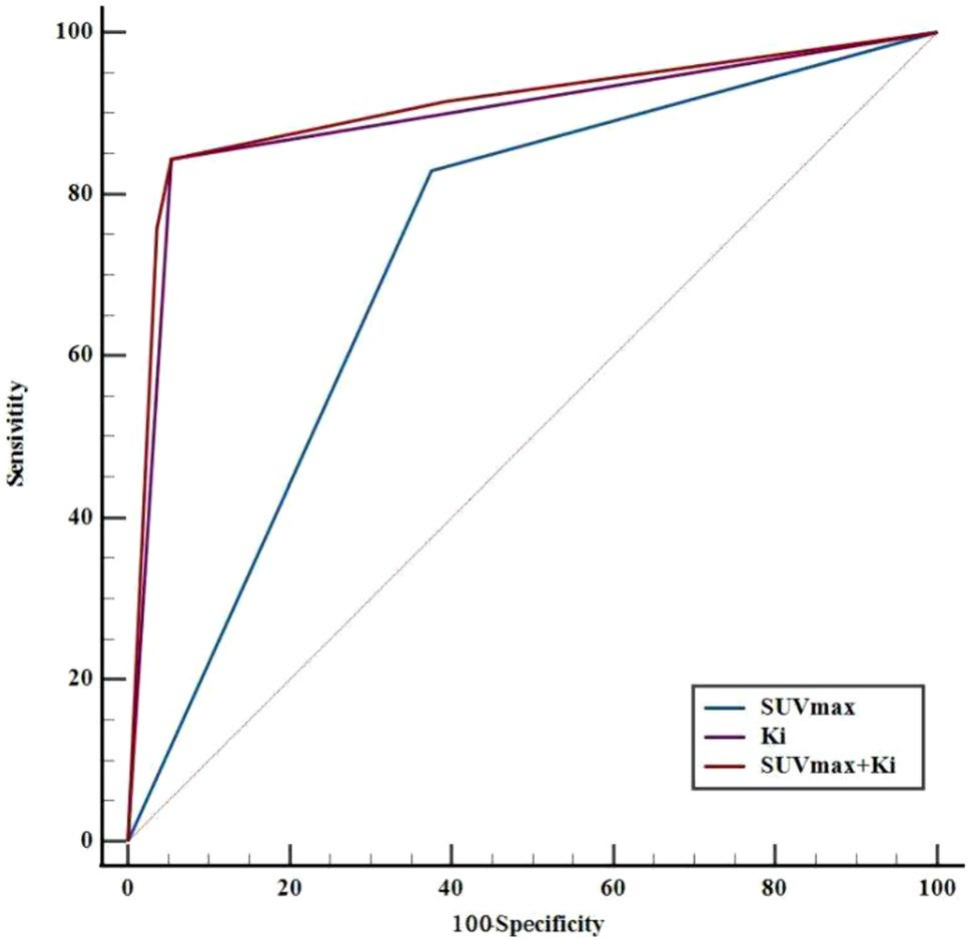
The results of the ROC analysis of SUV_max_ and *K*_i_ when tested individually and in combination

In previous studies, we have concluded that the cutoff values of 4.05, 0.236, 0.022 ml/g/min, and 0.093 for the static metabolic parameters SUV_max_, LN-SUV_max_/PT-SUV_max,_ and the dynamic metabolic parameters *K*_i_, *K*_i_/*K*_1_, respectively. Therefore, based on the above thresholds, we tested static metabolic parameters, and dynamic metabolic parameters individually and in combination. Table 2 and Fig. 2 show the results of ROC analysis of static and dynamic metabolic parameters when tested individually and in combination, respectively.

**Table 2:** ROC analysis results for each metabolic parameter when tested individually and in combination

Diagnostic methods	Pathology result		Total	SEN (%)	SPE (%)	PPV (%)	NPV (%)	Accuracy (%)	AUC (95% CI)
	Metastatic	Non-metastatic							
*SUV*_*max*_									
Metastatic	58	21	79	82.90	62.50	82.86	65.50	74.60	0.727 (0.640–0.802)
Non-metastatic	12	35	47						
Total	70	56	126						
*LN-SUV*_*max*_*/PT-SUV*_*max*_									
Metastatic	67	46	113	95.70	17.90	95.71	17.86	61.11	0.567 (0.477–0.656)
Non-metastatic	3	10	13						
Total	70	56	126						
*K*_*i*_									
Metastatic	59	3	62	84.30	94.60	84.29	94.64	88.89	0.895 (0.827–0.942)
Non-metastatic	11	53	64						
Total	70	56	126						
*K*_*i*_*/K*_*1*_									
Metastatic	59	19	78	84.3	66.1	84.29	66.07	76.19	0.752 (0.667–0.824)
Non-metastatic	11	37	48						
Total	70	56	126						
*SUV*_*max*_* + K*_*i*_									
Metastatic	59	3	62	84.3	94.6	84.29	94.64	88.89	0.907 (0.842–0.951)
Non-metastatic	11	53	64						
Total	70	56	126						
*SUV*_*max*_* + K*_*i*_*/K*_*1*_									
Metastatic	59	19	78	75.7	82.1	84.29	66.07	76.19	0.813 (0.734–0.877)
Non-metastatic	11	37	48						
Total	70	56	126						
*LN-SUV*_*max*_*/PT-SUV*_*max*_* + K*_*i*_									
Metastatic	59	3	62	84.3	94.6	84.29	94.64	88.89	0.901 (0.835–0.947)
Non-metastatic	11	53	64						
Total	70	56	126						
*LN-SUV*_*max*_*/PT-SUV*_*max*_* + K*_*i*_*/K*_*1*_									
Metastatic	59	19	78	84.3	66.1	84.29	66.07	76.19	0.756 (0.672–0.828)
Non-metastatic	11	37	48						
Total	70	56	126

In separate assays, the dynamic metabolic parameter *K*_i_ [sensitivity (SEN) of 84.30%, specificity (SPE) of 94.60%, positive predictive value (PPV) of 84.29%, negative predictive value (NPV) of 94.64%, accuracy of 88.89%, and AUC of 0.895] had a better diagnostic value than the static metabolic parameter SUVmax (SEN of 82.90%, SPE of 62.50%, PPV of 82.86%, NPV of 65.50%, accuracy of 74.60%, and AUC of 0.727) in differentiating between metastatic and non-metastatic LNs groups, respectively.

In the combined diagnosis group, the combined SUV_max_ + *K*_i_ diagnosis had a better diagnostic value in the differential diagnosis of metastatic from non-metastatic LNs, with SEN, SPE, PPV, NPV, accuracy, and AUC of 84.3%, 94.6%, 84.29%, 94.64%, 88.89%, and 0.907, respectively.

### Accuracy of metabolic parameters SUV_max_ and *K*_i_ in N staging

**Fig. 3:**
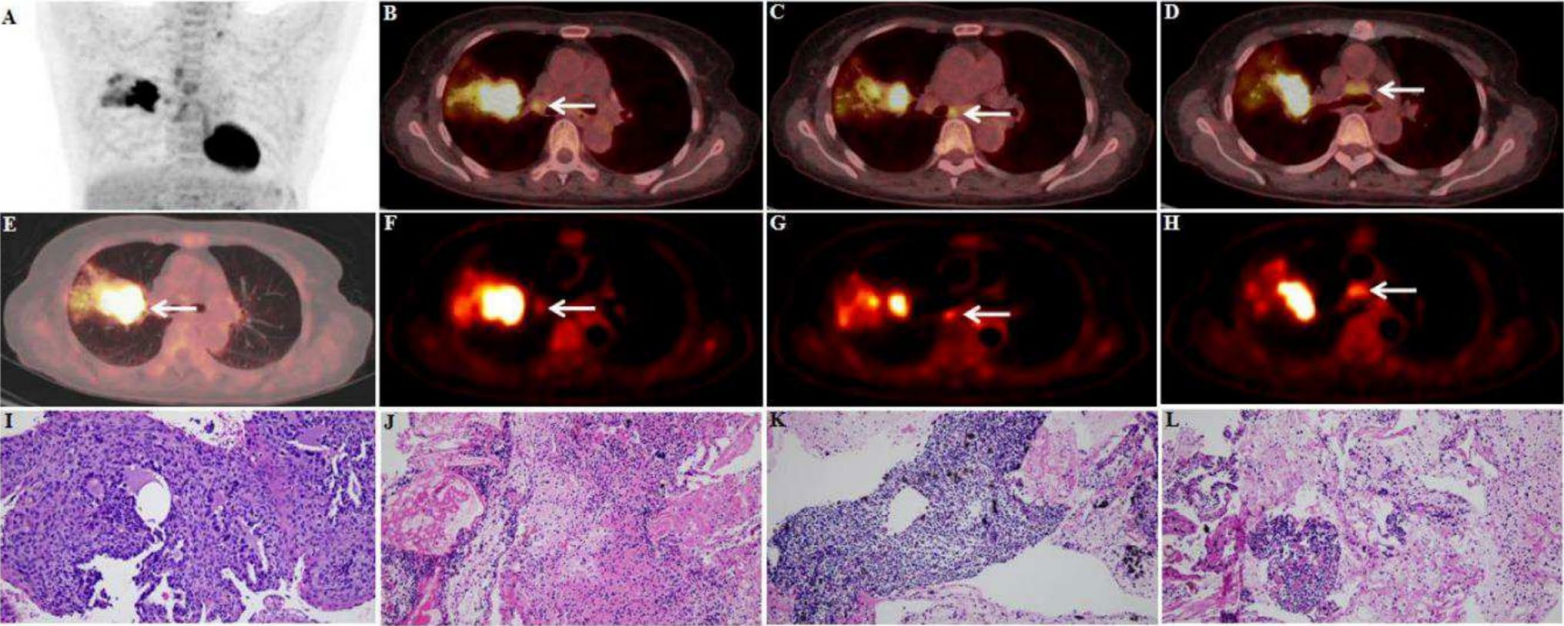
dPET/CT and sPET/CT images of non-metastatic FDG-avid LNs

Of the 126 LNs in 37 patients with lung cancer, 70 were metastatic, and 56 were non-metastatic. At the time of sPET/CT diagnosis, N-staging was consistent with pathology in 20 patients, but not in 17 patients, of which 15 patients were over-staged and 2 patients were under-staged. For the combined SUV_max_ + *K*_i_ diagnosis, N-staging was accurate in 30 patients, but N-staging was inconsistent with pathology findings in 7 patients, of which 3 patients were over-staged and 4 patients were under-staged. Table 3 shows the comparison of N-staging based on SUV_max_ and SUV_max_ + *K*_i_ diagnosis with pathological findings (Fig. 3).

**Table 3:** Accuracy of sPET/CT and dPET/CT N-staging in 37 patients with lung cancer

Pathology result	SUV_max_				SUV_max_ + K_i_			
	N0 (*N* = 4)	N1 (*N* = 1)	N2 (*N* = 8)	N3 (*N* = 24)	N0 (*N* = 12)	N1 (*N* = 5)	N2 (*N* = 6)	N3 (*N* = 14)
N0 (*N* = 13)	3	1	4	5	10	1	2	0
N1 (*N* = 3)	1	0	1	1	1	2	0	0
N2 (*N* = 5)	0	0	2	3	1	0	4	0
N3 (*N* = 16)	0	0	1	15	0	2	0	14

**Fig. 4:**
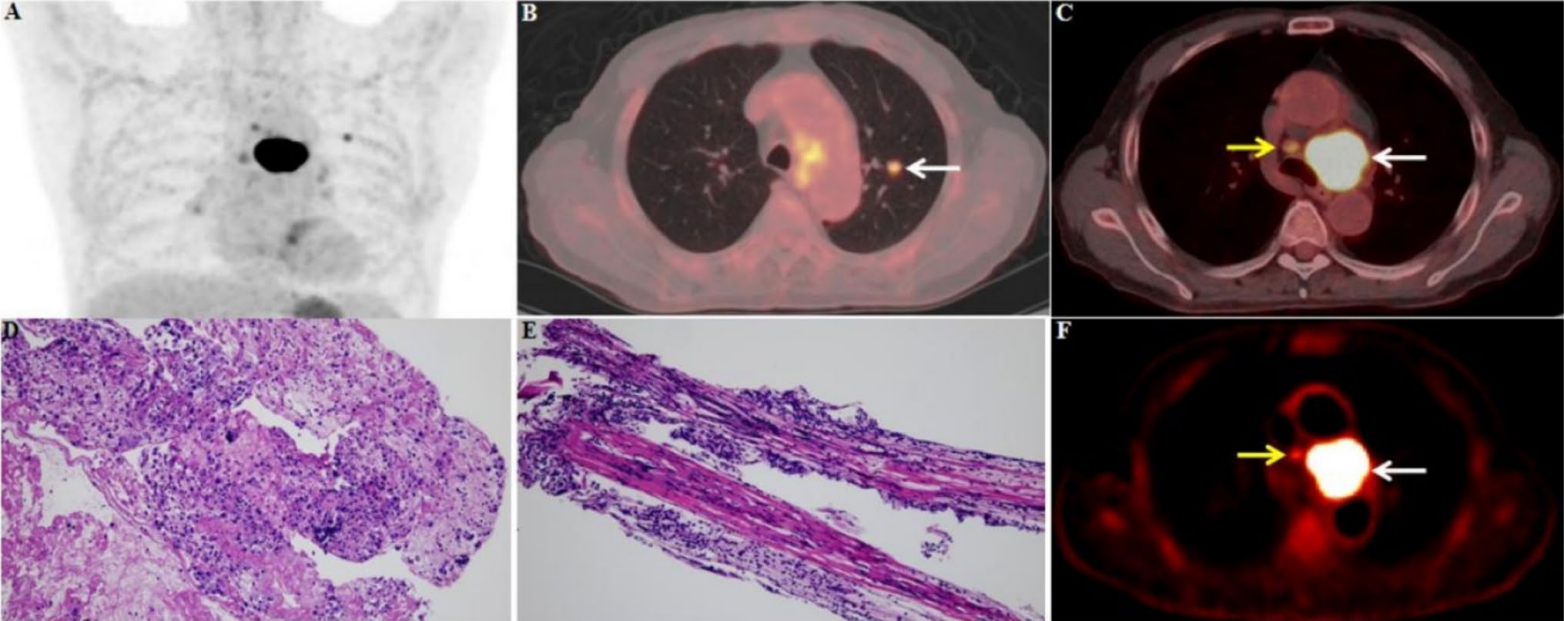
dPET/CT and sPET/CT images of non-metastatic and metastatic FDG-avid LNs

A 66-year-old female patient. Surgical pathology confirmed squamous cell carcinoma in the upper lobe of the right lung (A, E, I, white arrow, size of 3.5 × 3.1 cm, SUV_max_ of 14.8). FDG PET/CT scan showed multiple FDG-avid LNs in the zone 11R, 7, and 4R. Among them, the FDG-avid LN in zone 11R (B, F, white arrow) with a size of 1.4 × 1.3 cm, SUV_max_ of 4.0, and *K*_i_ of 0.010 ml/g/min. The FDG-avid LN in zone 7 (C, G, white arrow) with a size of 1.0 × 0.8 cm, SUV_max_ of 4.4, and *K*_i_ of 0.016 ml/g/min. The FDG-avid LN in zone 4R (D, H, white arrow) with a size of 2.1 × 1.3 cm, SUV_max_ of 4.1, and *K*_i_ of 0.013 ml/g/min. Finally, all FDG-avid LNs in zones 11R, 7, and 4R were pathologically confirmed to be cancer-free (Fig. 4).

A 66-year-old male patient. FDG PET/CT showed a nodule in the upper lobe of the left lung (A, B), with a size of 1.0 × 0.6 cm, and SUV_max_ of 4.8. FDG PET/CT scan showed FDG-avid LNs in zones 4L and 4R. Among them, the FDG-avid LN in zone 4L (C, F, white arrow) was pathologically confirmed adenocarcinoma metastasis (D), with a size of 4.2 × 3.8 cm, SUV_max_ of 34.6, and *K*_i_ of 0.135 ml/g/min. The FDG-avid LN in zone 4R (C, F, yellow arrow) was pathologically confirmed non-metastasis (E), with a size of 1.3 × 0.9 cm, SUV_max_ of 4.0, and *K*_i_ of 0.015 ml/g/min.

## Discussion

In lung cancer, accurate N-staging is essential for developing personalized treatment plans and determining prognosis and is one of the clinical concerns. In this study, based on the previous study, we further confirmed the high specificity of the dynamic metabolic parameter *K*_i_ in the differential diagnosis between metastatic from non-metastatic FDG-avid LNs in lung cancer. The combination of SUV_max_ and *K*_i_ is expected to be a reliable imaging basis for accurate N-staging of lung cancer.

Radiopharmaceutical distribution is a dynamic process that varies widely in diseases and individuals [20]. Previous studies have confirmed that dPET/CT extracts physiological and biochemical parameters that better reflect the pathophysiological mechanisms of disease compared to sPET/CT. These parameters (e.g., *K*_i_) have been shown to discriminate between benign and malignant diseases [10–15, 19, 21–23]. Previous studies have confirmed the value of dPET/CT in the differential diagnosis of lung cancer and inflammatory lesions, but there are fewer studies in the differential diagnosis of LNs. Previously, in our studies related to dynamic metabolic parameters in the differential diagnosis of lung cancer LNs [12], we discussed the value of dynamic metabolic parameters (including *K*_1_, *K*_2_, *K*_i_, and *K*_i_/*K*_1_) in the N-staging of lung cancer. We concluded that when the cutoff values of *K*_i_ and *K*_i_/*K*_1_ were 0.022 ml/g/min and 0.093, respectively, there was a good diagnostic value in the differential diagnosis of metastasis and non-metastasis in FDG-avid LNs of lung cancer, especially improving the specificity (AUC of 0.672 and 0.673, and specificity of 0.918 and 0.776, respectively). Therefore, to further investigate the diagnostic value and feasibility of dynamic metabolic parameters in N-staging of lung cancer based on the critical values of our previous study, we conducted this validation study.

In this study, we found that the static parameters SUV_max_ (cutoff value of 4.05) and LN-SUV_max_/PT-SUV_max_ (cutoff value of 0.236) had high sensitivity (82.5% and 92.7%) and accuracy (74.60% and 61.11%), but the specificity was not satisfactory (62.50% and 17.90%). The addition of the dynamic metabolic parameter K_i_ (cutoff value of 0.022) resulted in a significant improvement in the differential diagnosis when SUV_max_ and *K*_i_ were combined (AUC of 0.907), with high sensitivity (84.30%), specificity (94.60%), and accuracy (88.89%).

Among the 37 lung cancer patients in our study, at the time of sPET/CT diagnosis, N-staging was consistent with pathology in 20 (54.05%) patients, but not in 17 (45.94%) patients, of which 15 (40.54%) patients were over-staged and 2 (5.41%) patients were down-staged. For the combined SUV_max_ + *K*_i_ diagnosis, N-staging was accurate in 30 (81.08%) patients, but N-staging was inconsistent with pathology findings in 7 (18.92%) patients, of which 3 (8.10%) patients were over-staged and 4 (10.81%) patients were down-staged. After adding the dynamic metabolic parameter *K*_i_ for co-diagnosis, eight patients (21.62%) diagnosed as stage N3 by SUV_max_ were downgraded to stage N0–2, and four (30.77%) patients underwent successful surgical treatment. Therefore, SUV_max_ + *K*_i_ combined diagnosis has high diagnostic value in the differential diagnosis of FDG-avid LN metastasis from non-metastasis in lung cancer, especially to improve the specificity. In particular, it improves the diagnostic accuracy of patients with clinical suspicion of stage N3, seeks surgical opportunities as much as possible, and reduces some unnecessary puncture biopsies. To assist clinicians in accurate N-staging to improve the prognosis and quality of life of lung cancer patients.

Previous studies have reported that [24–26], the false-positive rate of squamous cell carcinoma on sPET/CT is significantly higher than that of adenocarcinoma and small cell carcinoma. This may be because squamous cell carcinomas are mostly central bronchogenic carcinomas, often associated with obstructive pneumonia and atelectasis, which can activate macrophages and inflammatory cells and cause reactive hyperplasia of LNs, leading to false-positive sPET/CT results [27]. In our sample size of 21 LNs (21/126) from 5 patients (5/37) with squamous cell carcinoma, the PT-SUV_max_ ranged from 8.5 to 16.4 and LN-SUV_max_ ranged from 2.5 to 11.5. N-staging sPET/CT failed to accurately stage all five patients, misstating two and over-staging three patients. Three of these patients were accurately staged with the SVU_max_ + *K*_i_ combination. Three (3/13) of these patients had pathologically confirmed N0, but SUV_max_ was assessed as N3 (1/3) and N2 (2/3), and with the addition of *K*_i_, the N3 (1/3)/N2 (1/3) stage was reduced to N0 (2/3). Among the patients in the adenocarcinoma group (*N* = 26), 17 patients (17/26) were accurately staged by sPET/CT, but 4 patients (4/26) were misstated and 5 patients (5/26) were over-staged. Among the over-staged patients (*N* = 5), PT-SUV_max_ ranged from 1.3 to 14.8, LN-SUV_max_ (*N* = 20) ranged from 2.6 to 17.2, and all were staged as stage N2 by sPET/CT. When SUV_max_ + *K*_i_ was combined with the test, the five patients who were over-staged as described above were accurately staged, with three down-staged to N0 and two down-staged to N1, all consistent with pathologic findings. Among patients with small cell carcinoma (*N* = 5), 3 patients (3/5) were accurately staged by sPET/CT, but 2 patients (2/5) were over-staged. Among the over-staged patients (*N* = 2), PT-SUV_max_ was 7.6 and 9.0, respectively, LN-SUV_max_ (*N* = 3) ranged from 2.6 to 4.2, and sPET/CT staging was N3 and N2, respectively. When SVU_max_ + *K*_i_ was detected in combination, the patients originally staged as N3 by sPET/CT were downgraded to N2, and those staged as N2 were downgraded to N0, which was consistent with the pathological results. The inclusion of the dynamic metabolic parameter *K*_i_ improved the specificity of the differential diagnosis. The combined SUV_max_ + *K*_i_ is expected to be a reliable metabolic parameter for lung cancer N-staging. In addition, whether the dynamic metabolic parameter *K*_i_ differs between pathology types and degree of differentiation is also of interest and one of our ongoing research topics.

There are several limitations to our study. First, we used a retrospective design due to our use of pathologic findings as the gold standard and the fact that the dPET/CT scan takes 65 min, resulting in our small sample size. In addition, prolonged scanning may lead to patient discomfort and feasibility issues. Second, motion correction was not considered in this study. It is known that motion in the chest region can affect not only the SUV but also the kinetic parameters quantification [28–30]. Third, SUV_max_ rather than SUV_mean_ was used in this study, because we believed SUV_max_ to be more stable and less affected by partial volume effects.

## Conclusions

When the cutoff value of the *K*_i_ was 0.022 ml/g/min, it had a high diagnostic value in the differential diagnosis between metastasis and non-metastasis in FDG-avid LNs of lung cancer, especially in improving the specificity. The combination of SUV_max_ and *K*_i_ is expected to be a reliable metabolic parameter for N-staging of lung cancer.

## Data Availability

The original contributions presented in the study are included in the article/supplementary material. Further inquiries can be directed to the corresponding authors.
